# Supplementary material for: The Nucleus Bypasses Obstacles by Deforming Like a Drop with Surface Tension Mediated by Lamin A/C
Source: Adv Sci (Weinh). 2022 Jun 16;9(23):2201248. doi: 10.1002/advs.202201248 (PMC9376816; doi:10.1002/advs.202201248)
Supplement: Supplementary file 1 — Supporting Information [file ADVS-9-2201248-s002.pdf]

ADVANCED SCIENCE
Open Access

Supporting Information

for *Adv. Sci.*, DOI 10.1002/advs.202201248

The Nucleus Bypasses Obstacles by Deforming Like a Drop with Surface Tension Mediated by Lamin A/C

Aditya Katiyar, Jian Zhang, Jyot D. Antani, Yifan Yu, Kelsey L. Scott, Pushkar P. Lele, Cynthia A. Reinhart-King, Nathan J. Sniadecki, Kyle J. Roux, Richard B. Dickinson and Tanmay P. Lele**

Supporting information

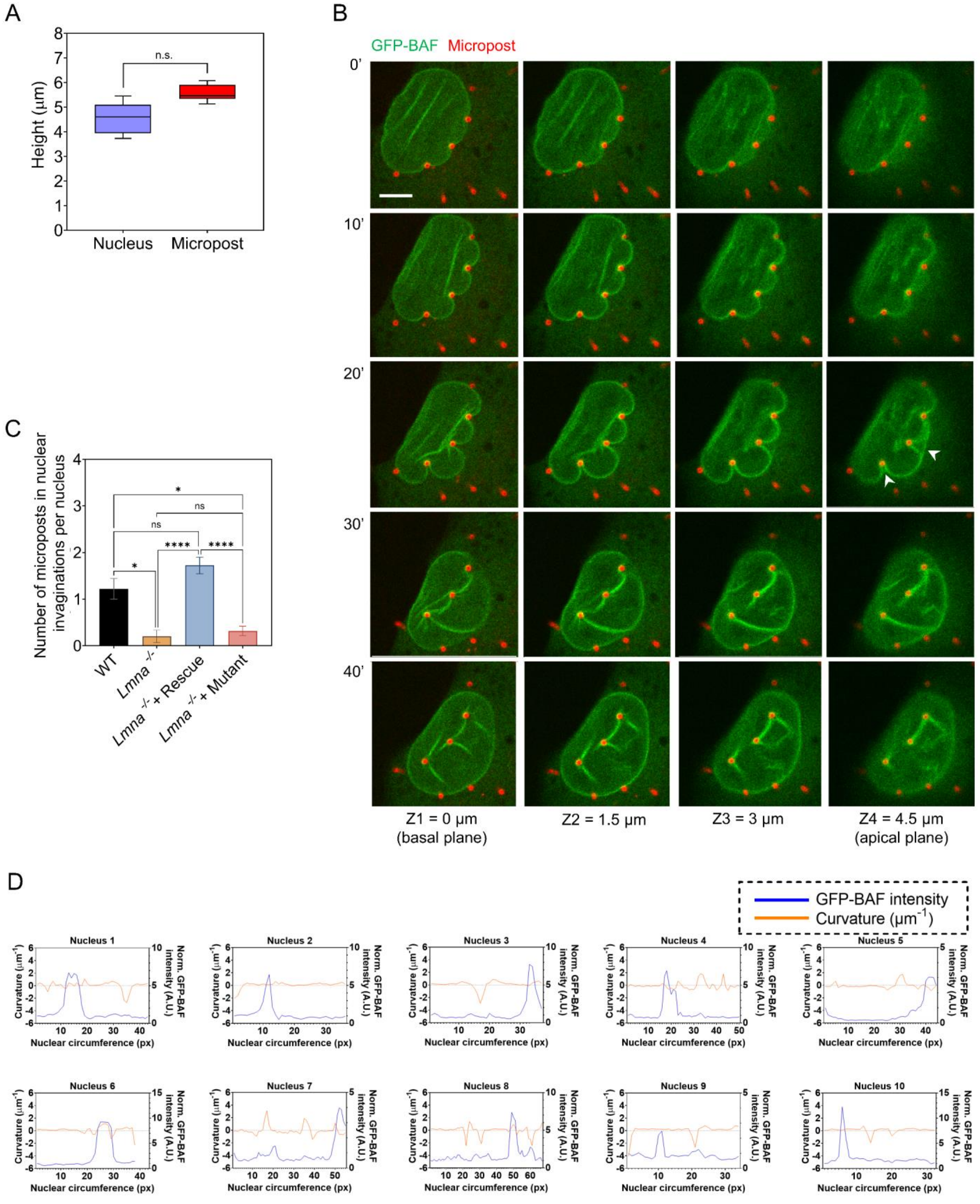

Figure S1: (A) Comparison of nuclear height measured in nuclei deformed around individual microposts with the height of microposts measured from reconstructed z-stacks; n ≥ 20 nuclei and n ≥ 20 microposts from at least three independent experiments. (ns $p > 0.05$, Student's *t*-

test). (B) Time-lapse confocal images of an NIH 3T3 fibroblast stably expressing GFP-BAF deforming around 5 µm tall rhodamine-fibronectin stained PDMS microposts (red) sliding over the top of the microposts (white arrowheads), without collapsing the vertical microposts, (Scale bar is 5 µm). (C) Box plot shows the number of microposts in nuclear invaginations by the nucleus per cell in MEF WT (n = 12 cells), MEF *Lmna*$^{-/-}$ (n = 18 cells), MEF *Lmna*$^{-/-}$ + GFP-Lamin A (rescue) (n = 22 cells) and MEF *Lmna*$^{-/-}$ + GFP-Lamin A (S22A/S392A) mutant (n = 25 cells) from three experiments for each condition. (* $p < 0.05$; Mann-Whitney test). (D) Plots of nuclear envelope curvature (orange curve) and GFP-BAF intensity (blue curve) along the circumference of nuclei (n = 10 cells) that deformed and underwent nuclear envelope rupture around microposts.

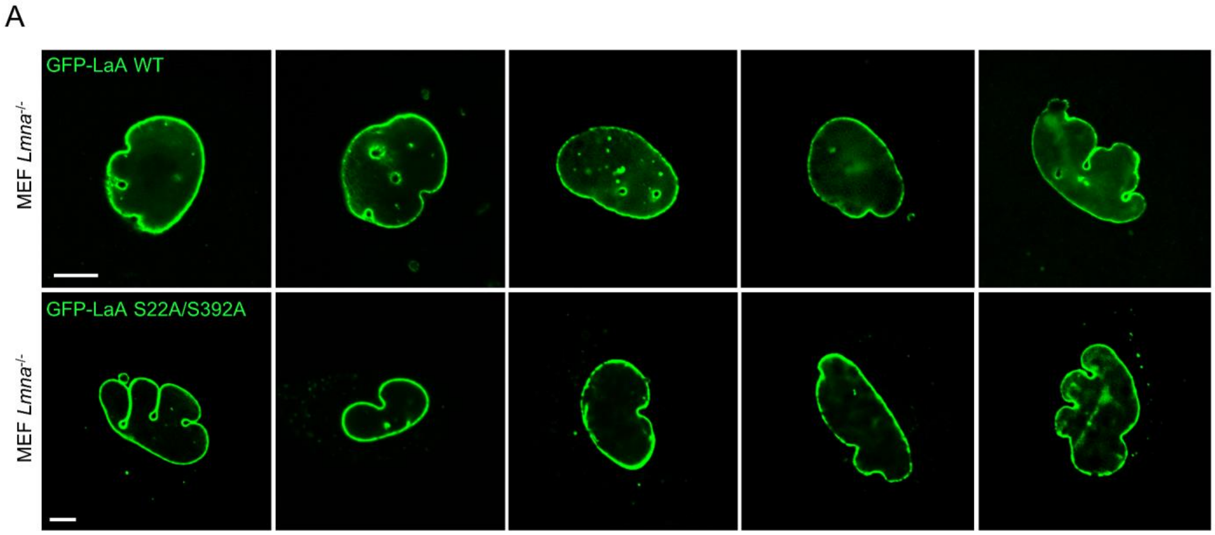

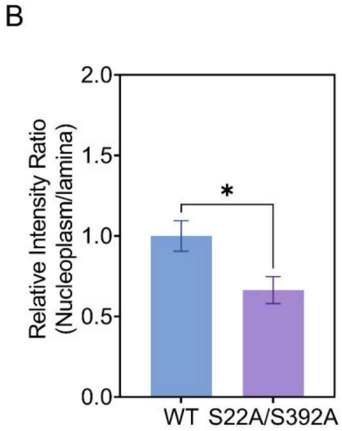

Figure S2: (A) Images of *Lmna*$^{-/-}$ nuclei expressing GFP-Lamin A WT in the top panel and GFP-Lamin A S22A/S392A in the bottom panel. (B) Bar graph compares nucleoplasmic to lamina intensity ratios of GFP-Lamin A (WT) and GFP-Lamin A S22A/S392A mutants (S22A/S392A), respectively. Data represents mean±SEM (n = 10 cells for each condition from three independent experiments, *: $p<0.05$, unpaired Student's *t*-test) normalized to the mean nucleoplasmic to lamina ratio of GFP-Lamin A.

Movies:

Movie 1: **Encounters of a GFP-BAF expressing cell with 5 µm PDMS microposts.** Time-lapse movie shows the nucleus of a GFP-BAF expressing fibroblast (green) moved unimpeded past the rhodamine-fibronectin labeled microposts (red) as contact with each new obstacle created a transient deep, local invagination in the nuclear surface (Scale bar is 5 µm).

Movie 2: **Encounters of a GFP-BAF expressing cell with 11 µm Si microposts.** Time-lapse movie shows deep invaginations developing in the nucleus of a GFP-BAF expressing fibroblast (green); the invaginations were separated by lobes of nearly constant curvature that allowed the nucleus to move unimpeded past the microposts (Scale bar is 10 µm).

Movie 3: **Examples of nuclei deforming around single collagen fibers in collagen gels.** Time-lapse movies of nuclei of GFP-BAF expressing fibroblasts deforming and wrapping around single collagen fibers in 3-D collagen gels or squeezing in-between the interstitial spaces between fibrils (Scale bar is 5 µm).

Movie 4: **Entanglement of *Lmna*$^{-/-}$ nuclei around microposts.** Time-lapse movie of MEF *Lmna*$^{-/-}$ nuclei stained with NucSpot Live 650 (a live-nuclear imaging dye, white) as they deform against rhodamine-labeled microposts (red) (Scale bar is 5 µm).

Movie 5: **WT nuclei bypassed the microposts by forming deep local invaginations while preserving the overall nuclear shape.** Time-lapse movie of MEF WT nuclei stained with NucSpot Live 650 (a live-nuclear imaging dye, white) as they deform against rhodamine-labeled microposts (red) (Scale bar is 5 µm).

Movie 6: **WT deformation behavior is rescued upon expression of GFP lamin A in *Lmna*$^{-/-}$ cells.** Time-lapse movie of an MEF *Lmna*$^{-/-}$ nucleus expressing GFP-Lamin A (green) deforming

against a rhodamine-fibronectin labeled micropost (red) and moving past it (corresponding to Figure 6C, third panel) (Scale bar is 10 µm).

Movie 7: **GFP-Lamin A (S22A/S392A) expression in *Lmna*$^{-/-}$ cells rescued WT deformation behavior but did not prevent entanglement.** Time-lapse movie of a MEF *Lmna*$^{-/-}$ nucleus (corresponding to Figure 6C, fourth panel) transfected with GFP-Lamin A (S22A/S392A) (green) and stained with NucSpot Live 650 (for DNA, blue), deforming around two rhodamine-fibronectin labeled microposts (red); white arrowheads show nuclear blebbing followed by NE rupture, as suggested by a decrease in cross-sectional area of the nucleus (Scale bar is 10 µm).

Movie 8: **Oil drop with higher surface tension deforming with a narrow, local invagination around the indenting wire.** The movie shows the behavior of an oil drop (blue) in water (yellow) when deformed with a thin metal wire (*diameter* = 0.5 mm) (Scale bar is 5 mm).

Movie 9: I**ndentation with a metal wire of the oil drop with a lower surface tension causes a more extreme shape distortion from the starting oval shape.** Time-lapse movie shows the behavior of an oil drop (yellow) with 3% (w/v) Triton X-100 in water when deformed with a thin metal wire (*diameter* = 0.5 mm) (Scale bar is 5 mm).
